# Inflammatory Bowel Disease in Hispanics: The University of Puerto Rico IBD Registry

**DOI:** 10.1155/2012/574079

**Published:** 2011-12-11

**Authors:** Esther A. Torres, Abdiel Cruz, Mariola Monagas, Marina Bernal, Yadira Correa, Rafael Cordero, Víctor L. Carlo

**Affiliations:** ^1^UPR Center for Inflammatory Bowel Disease, Department of Medicine, UPR School of Medicine, San Juan, PR 00936-5067, USA; ^2^Department of Ophthalmology, UPR School of Medicine, San Juan, PR 00936-5067, USA; ^3^UPR School of Medicine, San Juan, PR 00936-5067, USA

## Abstract

A registry of patients with inflammatory bowel diseases, ulcerative colitis (UC) and Crohn's disease (CD), was created at the University of Puerto Rico in 1995. Subjects with a documented diagnosis of IBD by clinical, radiologic, endoscopic, and/or pathologic criteria were recruited from the IBD clinics, support groups, and community practices, and demographic and medical data was collected. All entries from 1995 to 2009 were analyzed for demographics, family history, disease extent, extraintestinal manifestations, surgery, and smoking history. Results were described using summary statistics. 635 Hispanics living in Puerto Rico, 299 with UC and 336 with CD, were included. Mean ages were 40.3 for UC and 30.9 for CD. Over half (56%) of UC and 41% of CD were females. Family history was present in 19.3% of UC and 17.5% of CD. Surgery for IBD had been performed in 31.9% of UC and 51.2% of the CD patients. Over one-fourth of the patients reported extraintestinal manifestations, most frequently arthropathies. Our findings contribute to the limited epidemiologic and clinical data on Hispanics with IBD.

## 1. Introduction

The study of Crohn's (CD) and ulcerative colitis (UC) has been focused on Caucasians, as the incidence and diagnosis of inflammatory bowel disease (IBD) have been more evident in this population. The rates of UC and CD are highest in northern climates, in urban regions, and in well-developed areas of the world such as North America and Europe and lowest in southern climates and underdeveloped areas [1–4]. The reported prevalence of IBD in adults in the United States (US) has ranged from 37 to 238 per 100,000 for UC and 26 to 201 per 100,000 for CD [4, 5].

The epidemiology of IBD seems to be changing. Incidence in North America, Northern and Western Europe has stabilized, and low incidence areas are showing an increase [4]. The racial and ethnic differences also seem to be narrowing. Reports of IBD from Barbados and the French West Indies in the Caribbean illustrate these changes [6, 7].

There is an increasing recognition of IBD in the minority populations of the United States [7–16], and several studies have described the epidemiology of IBD in these populations. Many of these studies have centered on blacks with IBD, while IBD in Hispanics is less well defined. The incidence and prevalence of IBD in Puerto Rico has been rising over the past decades [17–19]. With the aim of further examining Hispanics with IBD, we describe the demographic and clinical characteristics of a large university-based registry of IBD in Hispanics living in Puerto Rico.

## 2. Materials and Methods

The University of Puerto Rico IBD Registry was created in 1995 [17]. The Registry is a database of patients with UC and CD that collects demographic and medical information at the time of interview. Subjects with documented diagnosis of IBD by clinical, radiologic, endoscopic, and/or pathologic criteria are recruited from the IBD clinics, support group, and community practices and represent diverse areas of Puerto Rico. After informed consent, data is collected through an interview by trained investigators and medical records review. Information collected includes age, gender, diagnosis, age at onset of symptoms, age at diagnosis, urban or rural living, place of birth and parentage, family history of IBD, smoking history, extraintestinal manifestations, extent of disease, medications, and surgery. Method of diagnosis, including endoscopic procedures, imaging, pathologic specimens, and surgical findings, is obtained from the treating physician's and/or clinic medical record. The diagnosis has to be established or confirmed by a gastroenterologist. No followup is provided for updating data in the study. The database is entered into an Excel spreadsheet. The Registry has continuing Institutional Review Board approval from the University of Puerto Rico Medical Sciences Campus (protocol no. 1250195).

This report includes only Hispanics living in Puerto Rico. Hispanic subjects were identified by having both parents of Puerto Rican, Dominican, Venezuelan, or Cuban origin. Place of birth and childhood home are recorded in the Registry. Five subjects were excluded because of non-Hispanic origins (two from the United States, one from Nevis, one from Israel, and one with a German mother). The vast majority of subjects (585) were born in Puerto Rico of Puerto Rican parents. Forty-four were born in the United States, three in the Dominican Republic, three in Cuba, and one in Venezuela.

All entries from 1995 until 2009 were analyzed for demographics, family history, smoking history, disease extent, extraintestinal manifestations, and surgery. Disease duration at the time of inclusion in the Registry was calculated from the time of symptom onset and from the time of diagnosis. Medications used have been reported in a separate study [20]. Summary measures were used to describe the study group.

## 3. Results

Six hundred thirty-five Hispanic patients, 299 (47.1%) with UC and 336 (52.9%) with CD, were included in the Registry during the study period. The ages ranged from 10 to 85 years old, with the youngest recording age of diagnosis at 7 years old.  Table 1 shows the demographics, family history, and smoking history of the population included in this study. Patients with UC were older, predominantly female, and smoked less at the time of diagnosis, when compared to patients with CD. Mean age for UC patients was 40.3 ± 15.6 with mean ages at onset of 31 ± 14.4 and at diagnosis of 32.6 ± 14.4. Mean age for CD patients was 30.9 ± 12.2, with a mean age at onset of 24.7 ± 11.7 and at diagnosis of 26.8 ± 12.6. For ulcerative colitis, interval from onset was 9.3 years and interval from diagnosis was 7.7 years. For Crohn's disease, interval from onset was 6.2 years and interval from diagnosis was 4.1 years. More than half (56%) of UC patients were females, whereas 59% of CD patients were males. Nearly 12% of IBD patients were smokers at the time of diagnosis.

Family history of IBD was present in 19.3% of UC and 17.5% of CD patients. In the cohort with UC, there were 28 firstdegree relatives with UC and 4 with CD. An additional 27 other relatives had UC and 4 had CD. For patients with CD, 17 first-degree relatives (parents, siblings, or offspring) were affected with CD and 28 with UC. Twenty-six more remote relatives (including grandparents, aunts/uncles, cousins and nephews/nieces) had CD and 27 had UC.

At the time of inclusion in the UPR IBD Registry, the disease extent in subjects with UC was as follows: 13% proctitis, 16.2% proctosigmoiditis, 21% left side colitis, and 49.6% pancolitis. In subjects with CD, 54.4% had ileal disease, 47% had colon involvement, 15.6% had perianal disease, 6.7% had jejunal disease, and 1.1% had upper gastrointestinal tract involvement. 31.9% of UC and over half (51.2%) of CD patients reported having surgery for IBD at the time of the interview.

Extraintestinal manifestations, reported in 171 patients, are shown in Table 2. The most common were the various arthropathies, similar in both UC and CD, followed by erythema nodosum, which was more frequent in CD (1.6% in UC versus 6.8% in CD, *P* < 0.002). Osteoporosis was found more frequently in CD, though not statistically significant (14 of 20 cases). Ophthalmologic manifestations were grouped together, as they were infrequent.

## 4. Discussion

Incidence and prevalence studies for Puerto Rico have shown an increase in both ulcerative colitis and Crohn's disease since the late 1990s [17–19].

The clinical presentation of IBD in Hispanics has not been well studied. Most of the published studies focus on incidence and prevalence with limited descriptions of clinical characteristics. Studies in which Hispanics with IBD are compared with Caucasians and other ethnic or racial populations are limited by the small number of Hispanics included. A study of 148 patients with IBD seen from 1999 to 2003 compared 58 Whites, 54 African Americans, and 30 Mexican Americans. Family history was present in only 10% of Mexican Americans as compared to 33% of Whites. In those with ulcerative colitis, no differences in treatment and surgery were found [13]. In another study comparing the same three groups (H, AA, W), there was no difference between African Americans and Mexican Americans when separately compared to Whites in terms of intestinal manifestations of CD and UC, respectively. Among UC patients, Whites had significantly higher incidence than Mexican Americans of joint symptoms (*P* < 0.0001) and osteoporosis (*P* = 0.001). Whites had a stronger family history of IBD and colorectal carcinoma. All the Mexican Americans with UC who were tested had positive p-ANCA compared to only 40% of Whites (*P* = 0.033). Proctitis alone occurred in 32% of Whites, but only in 9% of Mexican Americans with UC (*P* = 0.022). In general, there were no significant differences in medical treatment, surgeries, or hospitalizations in the UC and in the CD groups. In summary, Mexican Americans with IBD differed significantly from other ethnic groups in the distribution of IBD subtypes, family history, and serological markers [10].

A recent large study from the United States involving 1,126 subjects with IBD, of which 830 were White, 169 were Puerto Rican Hispanics, and 127 were African Americans, reported that Hispanics were at higher risk of developing perianal disease and erythema nodosum. Among UC patients, Hispanics had more proximal disease extent. Hispanics with CD, but not UC, had lower prevalence of family history of IBD than Whites. Hispanics were more likely to have undergone bowel diversion for CD (22.4% versus 7.4%, *P* = 0.001) but had fewer total surgeries for abdominal CD than Whites. The number of surgeries for perianal CD was similar among all racial groups. At 5 years, the proportion of surgery-free CD patients was 68% for Whites and 60% for Hispanics. The median survival time free from surgery was 9.8 yr for Whites and 6.6 yr for Hispanics, with no significant increased risk of CD-related surgery for Hispanics compared to white subjects. Hispanics had a higher prevalence of surgery indicated for chronic refractory UC (85.7% versus 40.7%, *P* < 0.001). Furthermore, Hispanics had a considerably higher rate of colectomy for any indication (chronic disease, dysplasia, or fulminant colitis) than white subjects (32.3% versus 15.8%, *P* < 0.01) [7].

A comparison between our UPR IBD Registry and other Hispanic populations including Puerto Rico [7], Spain [21], Portugal [22], Huelva, Spain [23], Mexico [24], Panamá [25], and Argentina [25] is shown in Tables 3 (CD) and 4 (UC). It includes gender distribution, mean age, family history of IBD, and percent with extraintestinal manifestations. The comparison is limited by a number of small studies, the data reported for each population, and the period included in the study. As IBD incidence is considered to be increasing in Hispanics, earlier studies such as the one from Argentina and Panamá may not be representative of actual population characteristics. A systematic review of IBD in Asians, Hispanics, and African Americans published in 2009 concludes that, although the incidence is rising in Hispanics, the literature regarding IBD manifestations is very limited [26].

## 5. Conclusions

Variation in findings between different Hispanic groups may be a result of a changing epidemiology accompanying a rising incidence of IBD, with earlier studies being unable to detect an increase in family history or the true prevalence of extraintestinal manifestations, as well as an evolution in disease phenotype over time. Differences between whites and Hispanics reported in various studies are not consistent. These may be related to study design, small numbers of Hispanics in the studies, true genetic variation, and environmental influences. The limited inclusion of minorities in research studies, partly due to the low incidence of IBD at the time, but also possibly secondary to underrecruitment, poor interest in participation and referral bias, may also affect the results.

More studies are needed for a better characterization and comparison of epidemiology and clinical presentation of IBD in Hispanics. This knowledge may impact the therapeutic approach to these patients as well as the development of health care strategies to improve access and outcomes.

## Figures and Tables

**Table 1:** General characteristics of the Registry patients.

	UC	CD
*n*	299	336
Male : female (%)	44 : 56	59 : 41
Mean age	40.3 ± 15.6	30.9 ± 12.2
Mean age onset	31 ± 14.4	24.7 ± 11.7
Mean age dx	32.6 ± 14.4	26.8 ± 12.6
Family history IBD *n* (%)	58 (19.3%)	59 (17.5%)
Smoking at dx (%)	10%	13.7%

CD: Crohn's disease; UC: ulcerative colitis; IBD: inflammatory bowel disease.

**Table 2:** Extraintestinal manifestations.

	UC (%)	CD (%)	Total IBD (%)
EIM	78 (26)	93 (27.6)	171 (26.9)
All arthropathies	58 (19.4)	76 (22.8)	134 (21.1)
Peripheral arthropathy	48 (16.1)	62 (18.4)	110 (17.3)
Ankylosing spondylitis	2 (0.7)	1 (0.3)	3 (0.5)
Sacroiliitis	8 (2.6)	13 (3.8)	21 (3.3)
Erythema nodosum*	5 (1.6)	23 (6.8)	28 (4.4)
Pyoderma gangrenosum	6 (2)	3 (0.9)	9 (1.4)
Uveitis and/or episcleritis	5 (1.7)	10 (3)	15 (2.4)
Primary sclerosing cholangitis	3 (1.0)	2 (0.6)	5 (0.8)
Osteoporosis	6 (2.0)	14 (4.1)	20 (3.1)

*P < 0.002.

**Table 3:** Crohn's disease: comparison with other Hispanic populations.

	UPR Registry	PR (NIDDK-IBDGR) [7]	Spain [21]	Portugal [22]	Huelva, Spain [23]
*n*	336	106	635	1692	30
Male : female (%)	59 : 41		48 : 52	44 : 56	57 : 43
Mean age	30.9		33	31	
EIM (%)	27.6				36.7
Fam hx IBD (%)	17.5	16.6	15	5.8	33.3

**Table 4:** UC: comparison with other Hispanic populations.

	UPR Registry	PR (NIDDK IBDGRC) [7]	Mexico [24]	Panama [25]	Argentina [25] F	Huelva, Spain [23]
*n*	299	62	848	15	38	40
Male : female (%)	44 : 56		45 : 55	60 : 40	39 : 61	45 : 55
Mean age	40.3		31.3	38	45	
EIM (%)	26		41.5			12.5
Fam hx IBD (%)	19.3	14.5	6.7			12.5
